# Are non-responders in a quitline evaluation more likely to be smokers?

**DOI:** 10.1186/1471-2458-5-52

**Published:** 2005-05-23

**Authors:** Tanja Tomson, Catrine Björnström, Hans Gilljam, Asgeir Helgason

**Affiliations:** 1 Stockholm Center for Public Health, Tobacco Prevention, Box 175 33, 118 91 Stockholm, Sweden; 2 Department of Public Health Sciences, Karolinska Institutet, Norrbacka S2, 171 76 Stockholm, Sweden; 3 Department of Oncology-Pathology, Karolinska Institutet, Sweden

## Abstract

**Background:**

In evaluation of smoking cessation programs including surveys and clinical trials the tradition has been to treat non-responders as smokers. The aim of this paper is to assess smoking behaviour of non-responders in an evaluation of the Swedish national tobacco cessation quitline a nation-wide, free of charge service.

**Methods:**

A telephone interview survey with a sample of people not participating in the original follow-up. The study population comprised callers to the Swedish quitline who had consented to participate in a 12 month follow-up but had failed to respond. A sample of 84 (18% of all non-responders) was included. The main outcome measures were self-reported smoking behaviour at the time of the interview and at the time of the routine follow-up. Also, reasons for not responding to the original follow-up questionnaire were assessed. For statistical comparison between groups we used Fischer's exact test, odds ratios (OR) and 95% confidence intervals (CI) on proportions and OR.

**Results:**

Thirty-nine percent reported to have been smoke-free at the time they received the original questionnaire compared with 31% of responders in the original study population. The two most common reasons stated for not having returned the original questionnaire was claiming that they had returned it (35%) and that they had not received the questionnaire (20%). Non-responders were somewhat younger and were to a higher degree smoke-free when they first called the quitline.

**Conclusion:**

Treating non-responders as smokers in smoking cessation research may underestimate the true effect of cessation treatment.

## Background

Tobacco is one of the leading causes of the global burden of disease, [1] demanding effective intervention strategies [2]. Smoking cessation treatment is generally considered to be among the most cost-effective life saving interventions in the health system [3] and telephone helplines for smoking cessation (quitlines) have proved to be both effective and cost-effective [4-6].

In evaluation of smoking cessation programs including surveys and clinical trials the tradition has been to treat non-responders as smokers [7-12]. However, bias due to failure to respond to follow-up is seldom assessed [13] for the different types of interventions and presently no reliable data exists supporting the pre-judgement that non-responders are more likely than responders to be present smokers. The existing empirical data on difference in smoking between responders and non-responders is often based on public health surveys [10]. However, the possibility exists that non-responders in smoking cessation programs using a telephone quitline may differ from non-responders in general health surveys. Thus, to categorically classify non-responders as smokers may underestimate the true effectiveness of smoking cessation treatment [9]. To our knowledge no previous studies investigating telephone treatment for smoking cessation have assessed the effect of non-response on reported abstinence rates.

In the present study we compare abstinence rates and population characteristics in responders and non-responders included in an evaluation of the Swedish quitline and assess reasons for not responding to the original follow-up questionnaire.

## Methods

### Study population

**Figure 1:**
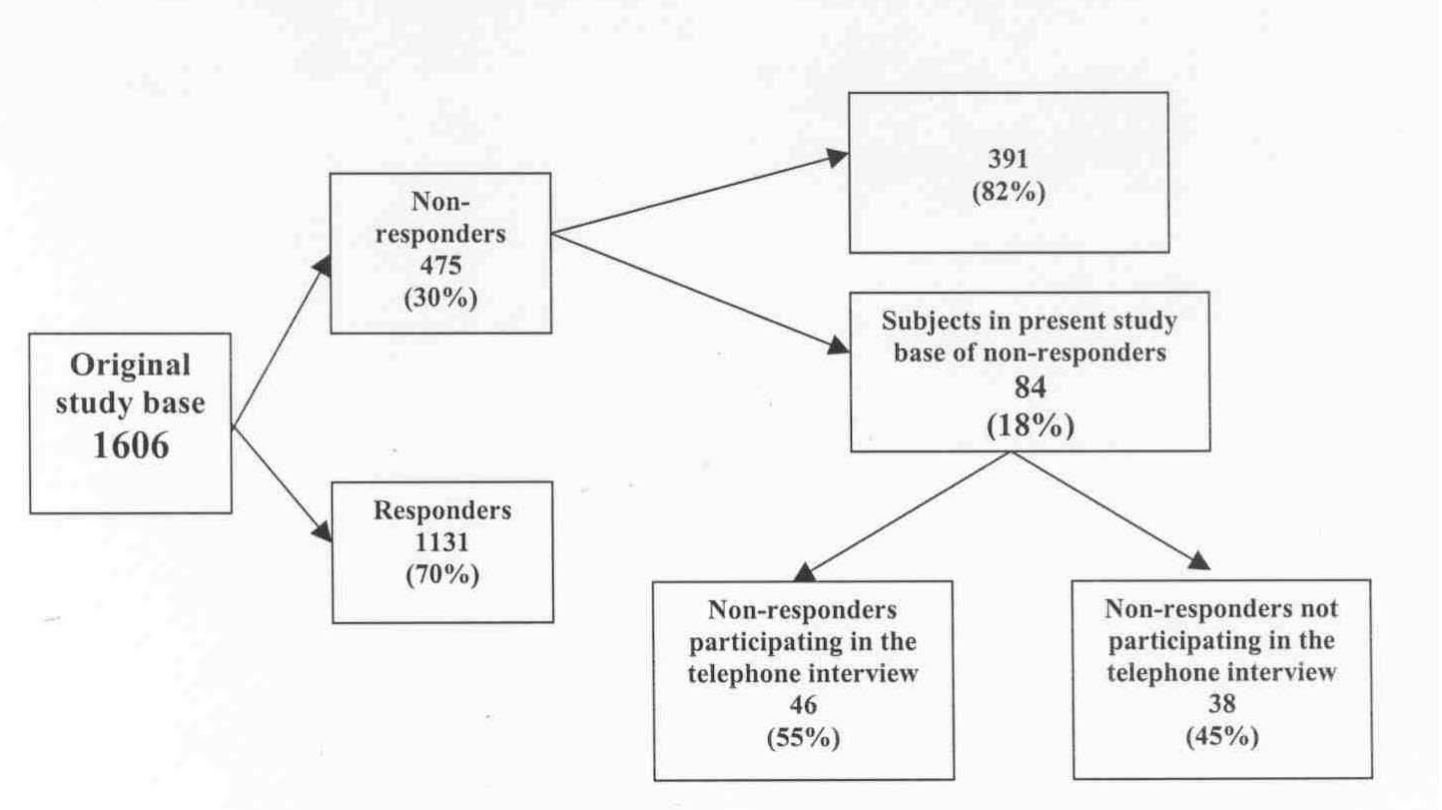
Graphic description of subjects in the study base.

The original study base included 1606 individuals in different stages of change who 12 months earlier had contacted the Swedish quitline for help with smoking cessation. All had accepted to receive a follow-up questionnaire 12 months after first contact with the quitline and participated in the original follow-up. First contacts were made from February 2000 to November 2001 and the follow-up questionnaires were mailed out February 2001 to November 2002. After two reminders answers were collected from 1131 individuals (70%) leaving 475 non-responders. A sample of 84 (18 % of the total non-response) was included in the present assessment of non-responders (Fig 1). In the beginning of November 2002 we identified all 84 patients who had not responded to the follow-up questionnaire during the previous four months. The relative narrow time window was to minimise recall bias.

### Data collection

The non-responders were contacted by a telephone interview during the autumn 2002. Each interview took approximately five minutes to perform and was conducted by only one person, to minimise interviewer bias. Attempts were made to phone first three times during daytime and then twice during evenings/weekends. Since all subjects had completed a questionnaire earlier (following their first call to the quitline) all background information, such as gender, age, language, tobacco habits and telephone number, was already available. In the original follow-up questionnaire abstinence was defined as "not a single puff of smoke during the previous seven days", which is a commonly used definition of point prevalence abstinence in larger surveys [12,14,15]. Two questions were used to assess present smoking behaviour: Have you smoked at least one single puff during the previous seven days? Has your smoking behaviour changed since you received the follow-up questionnaire? An additional retrospective question was asked to assess smoking behaviour at the time of the original 12 month follow-up: Have you smoked (one puff or more) in the week previous to receiving the follow-up questionnaire? The date when the original questionnaire was sent out was specified for all subjects. The interview was completed with a question on why he/she had not returned the original questionnaire. Ethical approval was obtained by Karolinska Institutet (Dnr 00-367).

### Statistical methods and presentation of data

We compared the prevalence of abstinence in the original study population (responders) with the 46 non-responders participating in the present study. In the table we start by comparing abstinence in the two groups at the time of the original follow-up and then at the time of the telephone interview.

Reasons stated by the subjects for not returning the original follow-up questionnaire are presented in a table as proportion of those 46 participating in the telephone interview. Reasons identified by the interviewer for not participating in the telephone interview are presented in the text as proportion of those 38 not participating.

Non-responders (at 12 month follow-up) participating in the telephone interview were compared with responders in the original study population in terms of population characteristics (sex, age, smoke-free at first call, and using nicotine at first call). These data are presented in table form. In the table we also present background data on all 84 non-responders selected for the study and the 38 non-responders not participating in the telephone interview. In the table we dichotomised age as a two category variable (≤40, ≥41), nicotine use at baseline as "yes" or "no" and stages of change as "still smoking at the time of first contact" and "smoke free at the time of first contact".

For statistical comparison between groups we calculated Fisher's exact test, odds ratios (OR) and 95% confidence intervals (CI) on proportions and OR. In table 1 we used one- sided CI on the proportions since our main focus was on the lower limits. Statistical analyses were carried out using the Statistical Package for the Social Sciences (SPSS 11.5).

**Table 1:** Percentage and proportions of abstinence in the original study population (responders) and the present study population (non-responders) at 12 months follow-up, and at the time of the telephone interview.

	**Original study population**	**The present study population participating in the telephone interview**	
	**Abstinent at 12 months**	**Abstinent at 12 months**	**Abstinent at the time of the tel. Interview**
	**% (n/N) One-sided 95%CI**	**% (n/N) One-sided 95%CI**	**% (n/N) One-sided 95%CI**

Men	30 (69/226)	63 (5/8)	38 (3/8)
	≥25	≥29	≥11
Women	31(285/905)	34 (13/38)	26 (10/38)
	≥29	≥22	≥15
**Total**	31 (354/1131)	39 (18/46)	28 (13/46)
	≥29	≥27	≥18

## Results

Of the 84 subjects not responding to the original questionnaire at 12 month follow-up (non-responders) recruited for the study base 55% (46/84) participated. Of the 38 subjects not participating 61% (23/38) could not be reached, 29% (11/38) declined, and 10% (4/38) were either sick or dead (not in table).

### Abstinence

Of the 46 subjects participating in the present study 39% reported to have been smoke-free at the time when they received the original follow-up questionnaire (abstinent at 12 months) compared with 31% of responders in the original study population (Table 1). No significant difference in abstinence was noted between the present study population and the original study population (Table 1). However, men in the present study population were somewhat more likely to report being abstinent at 12 months compared with the men in the original study population (Table 1). The reported higher level of 12 months abstinence in men did not persist at the time of the telephone interview (Table 1). One woman did not remember whether or not she was abstinent at twelve months and was treated as a smoker.

### Reasons for not returning the postal questionnaire

The most common reason stated for not having returned the original questionnaire was claiming that they had returned it (Table 2). Approximately one in ten stated that they had believed that abstinence was a prerequisite for answering and therefore had not returned the questionnaire since they were smoking at the time (Table 2).

**Table 2:** Stated reasons among 46 participants for not returning the postal questionnaire

	**% **	**(n)**
Claimed to have returned the questionnaire	35	(16)
Had not received the questionnaire/moved	20	(9)
Thought abstinence was a prerequisite for answering	13	(6)
Do not know	13	(6)
Forgot to return it	9	(4)
Uninterested	6	(3)
Had lost the questionnaire	4	(2)
**Total**	100	(46)

### Comparison with responders

The non-responders comprising the study base in the present study were somewhat younger than the responders in the original study population (Table 3). The mean ages being 47 for the responders and 42 for the non-responders (data not in table). Men and women were equally represented both among responders and non-responders. Non-responders tended to a higher degree to have been smoke-free when they first called the quitline (Table 3). They were also significantly more likely to have been totally nicotine free at first call compared with the responders (Table 3).

**Table 3:** Population characteristics of responding and non-responding subjects. Comparing 46 non-responders participating in the non-response analysis with the 1131 responders in the original study population.

**Total**	**Total sample of non-responders**	**Non-responders not participating in the telephone interview**	**Non-responders participating in the telephone interview**	**Responders in the original study**	**Comparison ¶**	
	**% (n) 100 (84)**	**% (n) 100 (38)**	**% (n) 100 (46)**	**% (n) 100 (1131)**	**OR**	**95%CI**
**Sex**						
**Male **(Ref)	20 (17)	24 (9)	17 (8)	20 (226)		
Female	80 (67)	76 (29)	83 (38)	80 (905)	1.2	0.5 – 2.6
**Age distribution**:						
≥ **41 **(Ref)	58 (49)	61 (23)	57 (26)	67 (755)		
≤ 40	42 (35)	39 (15)	43 (20)	33 (376)	1.5	0.9 – 2.8
**Smoke-free at first call:**						
**No **(Ref)	73 (61)	76 (29)	70 (32)	77 (875)		
Yes	27 (23)	24 (9)	30 (14)	23 (256)	1.5	0.8 – 2.8
Using nicotine* at first call:						
**Yes **(Ref)	82 (69)	87 (33)	78 (36)	89 (1010)		
No	18 (15)	13 (5)	22 (10)	11 (121)	2.3	1.1 – 4.8

*Total consumption of nicotine, including smoked and smoke-free tobacco and NRT.

¶Comparing non-responders participating in the telephone interview to responders

## Discussion

Our results indicate that non-responders in follow-ups of large cohorts of smokers trying to quit with the aid of telephone quitlines, should not by definition be considered as treatment failures.

If anything, the non-responders in the present study reported higher abstinence rates at the time when they were supposed to return the original follow-up questionnaire. The observed trend of overrepresentation of non-smokers among non-responders may be explained by the fact that they were more likely to be nicotine free at first call to the quitline. In the original follow-up we presented data separately for different stages of change. Those who were in the contemplation stage at first call reported 12-month abstinence in 19% of the cases compared with 22% for those in preparation and 53% for people who were in the action stage at first call [14]. Thus, being nicotine free at first call is a significant predictor for abstinence at 12 months. Our results indicate that classifying non-responders as smokers may underestimate the true treatment effect of quitlines in line with a recent Dutch study [17].

There is a tendency to view non-responders as a homogenous group with common characteristics but studies have not confirmed this to be the case [16]. A previous Swedish study comparing prevalence of smokers amongst non-responders to a general health survey with responders, showed a tendency for an overrepresentation of smokers amongst non-responders, especially in low income groups [10]. Contrary, a study from Holland did not find such differences [18]. However, caution is needed when comparing these studies to the present study since it is possible that non-responders in general health surveys may differ from non-responders in studies assessing abstinence rates after smoking cessation treatment.

Based on a response rate of approximately 70% in the original study, our results suggest that non-responders in the assessment of quitlines are probably not more likely than responders to be smokers at the time of follow-up. However, the possibility exists that non-responders in studies with lower response rate may differ from the present study population and our results may only apply for studies with a similar or higher response rate. The present results are in line with a study based on a one-year follow-up of participants from a national "Quit and Win" contest where bias in smoking prevalence because of non-response was studied [19].

Approximately one in three (29%) of those who were reached by telephone refused to participate in the interviews. There is a high probability that those individuals may be present smokers.

Methodological considerations include the relative low number of subjects compromising statistical power. The data indicate that non-responders are not more likely than responders to be smokers. However, owing to small numbers we are not able to conclude that the non-responders may be more likely to be smoke-free as the data indicates.

Another problem is the retrospective assessment of smoking behaviour at the time the non-responders were supposed to have turned in the questionnaire. Obviously recall bias may have affected the answers. A more conservative way to interpret the data is to compare the responders in the original study with non-responders smoking behaviour at the time of the telephone interview (Table 1, columns one and three). This more conservative comparison does not change the main results that using methods with no visual contact between counsellor and patient, non-responders may well fail to respond due to other reasons than active smoking.

As in all questionnaire surveys on smoking cessation the possibility of underreporting of smoking may exist e.g. due to the impact of social desirability (the desire to appear good). However, since this would most probably affect all subjects equally it is not a major concern in the present study. Also, a self-reported smoking status appears to be a reliable indicator of actual smoking-status [20] and the effect of social desirability in smoking cessation studies is probably less than previously suggested [21]. Further, in a recent study assessing if subjects who decline cotinine tests are lying about their smoking behaviour it was found that failure to comply may result from external factors such as demands or random factors such as being too busy at the time [22]. In the present study, thirteen percent stated that they thought abstinence was a prerequisite for returning the questionnaire (Table 2). This is a pedagogical problem that needs to be taken seriously since this may be a potential source of bias although not a major problem in the present study.

## Conclusion

The present study, one of the first of its kind, indicates that routinely treating non-responders as smokers in smoking cessation research may underestimate the true effect of cessation treatment.

## Competing interests

The author(s) declare that they have no competing interests.

## Authors' contributions

Tanja Tomson have made substantial contributions to conception and design, analysis and interpretation of data. She has been writing all drafts of this paper.

Catrine Björnström have contributed in the planning, design and analysis of this paper and have given final approval of the version to be published.

Hans Gilljam participated in the planning of the study design and assisted in the writing of the paper. He have given final approval of the version to be published

Asgeir Helgason have made substantial contributions to conception and design, analysis and interpretation of data. He has been involved in drafting the paper and have given final approval of the version to be published.

## Pre-publication history

The pre-publication history for this paper can be accessed here:


